# Hydrogen sulfide stimulates lipid biogenesis from glutamine that is dependent on the mitochondrial NAD(P)H pool

**DOI:** 10.1016/j.jbc.2021.100950

**Published:** 2021-07-10

**Authors:** Sebastian Carballal, Victor Vitvitsky, Roshan Kumar, David A. Hanna, Marouane Libiad, Aditi Gupta, Jace W. Jones, Ruma Banerjee

**Affiliations:** 1 Department of Biological Chemistry, Michigan Medicine, University of Michigan, Ann Arbor, Michigan, USA; 2 Departamento de Bioquímica, Facultad de Medicina and Centro de Investigaciones Biomédicas (CEINBIO), Universidad de la República, Montevideo, Uruguay; 3 Department of Pharmaceutical Sciences, University of Maryland School of Pharmacy, Baltimore, Maryland, USA

**Keywords:** bioenergetics, electron transport chain, hydrogen sulfide, lipid synthesis, lipidomic, cell metabolism, metabolic reprogramming, NAD(P)H, redox signaling, reductive carboxylation, DMEM, Dulbecco's modified Eagle's medium, DPBS, Dulbecco's phosphate-buffered saline medium, EMEM, Eagle's minimal essential medium, ETC, electron transport chain, FBS, fetal bovine serum, HCEC, human colonic epithelial cell, IDH, isocitrate dehydrogenase, MTBE, tert-butyl methyl ether, OCR, oxygen consumption rate, SQOR, sulfide quinone oxidoreductase, TCA, tricarboxylic acid cycle

## Abstract

Mammalian cells synthesize H_2_S from sulfur-containing amino acids and are also exposed to exogenous sources of this signaling molecule, notably from gut microbes. As an inhibitor of complex IV in the electron transport chain, H_2_S can have a profound impact on metabolism, suggesting the hypothesis that metabolic reprogramming is a primary mechanism by which H_2_S signals. In this study, we report that H_2_S increases lipogenesis in many cell types, using carbon derived from glutamine rather than from glucose. H_2_S-stimulated lipid synthesis is sensitive to the mitochondrial NAD(P)H pools and is enabled by reductive carboxylation of α-ketoglutarate. Lipidomics analysis revealed that H_2_S elicits time-dependent changes across several lipid classes, *e.g.*, upregulating triglycerides while downregulating phosphatidylcholine. Direct analysis of triglyceride concentration revealed that H_2_S induces a net increase in the size of this lipid pool. These results provide a mechanistic framework for understanding the effects of H_2_S on increasing lipid droplets in adipocytes and population studies that have pointed to a positive correlation between cysteine (a substrate for H_2_S synthesis) and fat mass.

Perturbations in the electron transport chain (ETC), which converts energy captured as reducing equivalents from oxidative metabolism into the currencies of ATP and membrane potential, have widespread effects on metabolism. Endogenous modulators of the ETC are therefore of interest as potential regulators of cellular metabolism, rendering it responsive to intrinsic as well as extrinsic cues. H_2_S is one such modulator and is derived from metabolism of the sulfur amino acids, cysteine and homocysteine (1). Despite a growing literature reporting varied cellular and physiological effects of H_2_S (2, 3), mechanistic insights into how H_2_S signals are limited (4).

Complex IV is a *bona fide* cellular target of H_2_S, explaining its long-known toxicity as an environmental poison (5). Steady-state concentrations of H_2_S are very low in most cell types and tissues (6, 7) and influenced by the kinetics of its synthesis and oxidation. Three enzymes, cystathionine β-synthase (8), γ-cystathionase (9), and mercaptopyruvate sulfur transferase (10) synthesize H_2_S, whereas enzymes in a mitochondrial resident pathway catalyze its oxidation to thiosulfate and sulfate (11). Cells can also be exposed to exogenous H_2_S particularly at the host–microbiota interface; gut microbial metabolism is estimated to expose colon epithelial cells to 0.2 to 2.4 mM H_2_S (12, 13). The reversibility of complex IV inhibition by H_2_S underlies its potential to modulate metabolism by perturbing mitochondrial bioenergetics (4).

Sulfide quinone oxidoreductase (SQOR) catalyzes the first step in the H_2_S oxidation pathway, forming glutathione persulfide (14, 15, 16). The latter is oxidized by ETHE1 to sulfite, releasing GSH (17). SQOR is a mitochondrial inner membrane protein that transfers electrons released during H_2_S oxidation to coenzyme Q and connects to the ETC at the level of complex III (18). Hence, H_2_S can both provide electrons to and inhibit the ETC, and SQOR plays a critical role as a respiratory shield, reducing exposure of complex IV to H_2_S (19). As SQOR is the committing enzyme in the sulfide oxidation pathway, regulation of SQOR expression levels and/or activity could be instrumental for transiently building up intracellular H_2_S levels. SQOR deficiency leads to increased sensitivity to H_2_S poisoning at a cellular level (19) and to Leigh’s disease in man (20).

Population and animal model studies have pointed to a role for cysteine and H_2_S in regulating lipid metabolism (21). Plasma total cysteine is positively correlated with obesity, specifically with fat mass (22). Of importance, this correlation is not general to amino acids including the other sulfur amino acids: methionine, homocysteine, and cystathionine (23). Although the underlying mechanism for this correlation is unknown, it has been speculated that cysteine regulates energy expenditure. Correlations between plasma H_2_S and adiposity have also been reported (24) but should be viewed with caution, owing to the technical difficulties with and lack of standardization of H_2_S measurements (reviewed in (21)). γ-Cystathionase knockout mice exhibit lower plasma total cysteine and reduced body weight and white adipose tissue (25). γ-Cystathionase is the second enzyme in the transsulfuration pathway and generates H_2_S from cysteine and/or homocysteine (9).

In a study on differentiated adipocytes, H_2_S was shown to increase the size and number of lipid droplets and to decrease lipolysis (26). The molecular mechanism by which H_2_S influences lipid metabolism is, however, not known. Oxidative metabolism of glucose and glutamine furnish citrate-derived acetyl-CoA for lipid biogenesis. Studies in our laboratory have demonstrated that H_2_S affects the metabolism of both glucose and glutamine in a manner that predicts opposite effects of these carbon sources on lipid synthesis. Thus, H_2_S stimulates aerobic glycolysis and leads to the stoichiometric conversion of glucose to two equivalents of lactate (27). On the other hand, by inducing a reductive shift in the NAD^+^/NADH ratio, H_2_S stimulates reductive carboxylation, *i.e.*, the conversion of glutamine-derived α-ketoglutarate to isocitrate (Fig. 1*A*) (19). These observations suggest the hypothesis that H_2_S reprograms energy metabolism and supports lipid biosynthesis using the glutamine-derived pathway for acetyl-CoA while simultaneously inhibiting β-oxidation by targeting complex IV.

**Figure 1:**
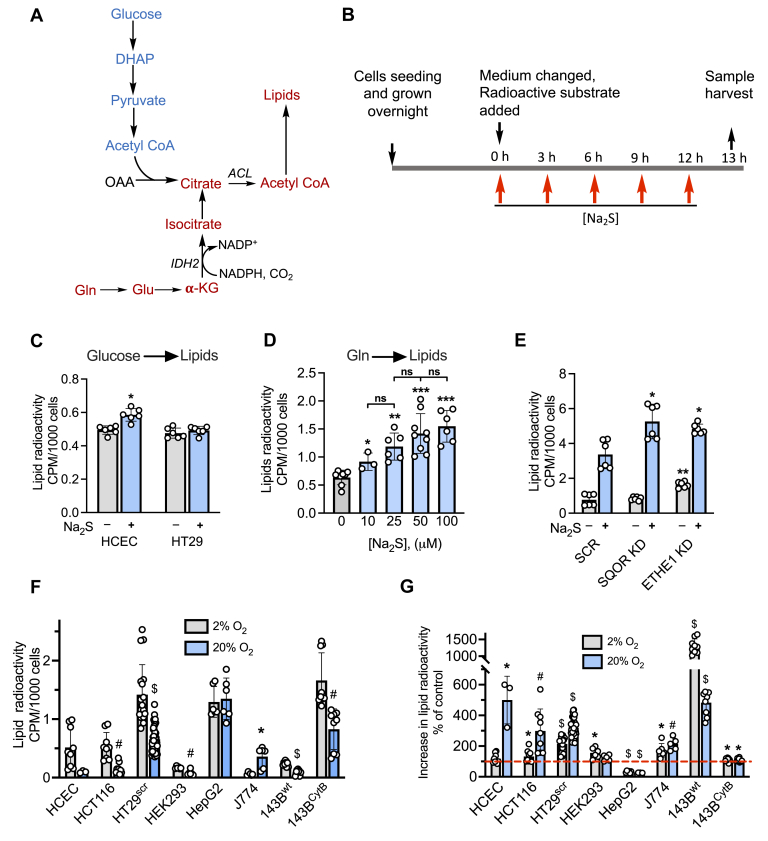
**H**_**2**_**S stimulates lipid biosynthesis from glutamine.***A*, pathways for labeling lipids from glucose (*blue*) and glutamine (*red*). IDH2 catalyzes the reductive carboxylation of α-KG (α-ketoglutarate), and ACL denotes ATP citrate lyase. *B*, scheme showing experimental setup used to monitor the effect of Na_2_S on lipid labeling from [U-^14^C]-glucose or [U-^14^C]-glutamine. The final concentration immediately following each addition of Na_2_S was 100 μM. *C*, radiolabeling of lipids from [U-^14^C]-glucose in human colonic epithelial cell and HT29 cells cultured in the presence or absence of Na_2_S. ∗Indicates statistically significant difference from control, *p* < 0.001. *D*, the effect of varying Na_2_S concentration (immediately following addition) on radiolabel incorporation into lipids in HT29^scr^ cells (∗*p* < 0.02, ∗∗*p* < 0.0002, ∗∗∗*p* < 0.0001 *versus* untreated control, ns indicates not significant). *E*, the effect of SQOR or ETHE1 knockdown in HT29 cells on lipid labeling. ∗, ∗∗ Indicate statistically significant (*p* < 0.005) differences from sulfide-treated control cells (HT29^scr^) and untreated controls, respectively. *F*, the effect of oxygen concentration on radiolabel incorporation from [U-^14^C]-glutamine into lipids in different cell lines. ∗^, #, $^Indicate significant differences between the adjacent bars with *p* < 0.003, 0.001, and 0.0001, respectively. *G*, O_2_ modulation of H_2_S-induced changes in metabolic labeling of lipids from [U-^14^C]-glutamine. The *red line* denotes the level of radiolabel incorporation in control cells, which was set at 100%. ∗^, #, $^Denote *p* < 0.05, <0.005, and *p* < 0.0001, respectively, *versus* untreated controls. Data represent mean ± SD (n = 3–41 independent experiments).

In this study, we report that H_2_S stimulates lipid synthesis from glutamine but not glucose, and that this response is seen across various malignant and nonmalignant cell lines. Of interest, metabolic flux from glutamine to lipids is sensitive to mitochondrial but not cytoplasmic NAD(P)H and is correlated with this pool affecting sulfide-stimulated oxygen consumption kinetics. Lipidomics analysis reveals that H_2_S elicits time-dependent changes across various classes of lipids. Collectively, these data reveal the ability of H_2_S to reprogram energy metabolism and impact lipid homeostasis.

## Results

### Sulfide stimulates lipid synthesis from glutamine

We examined the effect of sulfide on lipid biogenesis from [U-^14^C]-glucose or [U-^14^C]-glutamine in nonmalignant human colonic epithelial cell (HCEC) and malignant HT29 colorectal carcinoma cells (Fig. 1). We have previously demonstrated that, under cell culture conditions, H_2_S is lost from the growth medium in ∼30 min (27). To observe sufficient radiolabel incorporation into the lipid pool, exogenous sulfide (100 μM) was added every 3 h over a period of 12 h and samples were collected at t = 13 h (Fig. 1*B*). Sulfide resulted in a small (≤18%) but statistically significant increase in radiolabel incorporation from [U-^14^C]-glucose into lipids in HCEC cells but had no effect in HT29 cells (Fig. 1*C*). Since H_2_S stimulates reductive carboxylation in malignant colorectal cancer cells (19), lipids are predicted to be labeled by [U-^14^C]-glutamine (Fig. 1*A*). Indeed, sulfide elicited a significant increase in radiolabel incorporation from glutamine into lipids in control HT29^scr^ cells (transfected with a scrambled sequence), which was dependent on the concentration of sulfide added (Fig. 1*D*).

Next, we examined the effect of the sulfide oxidation enzymes SQOR and ETHE1 on the ability of sulfide to stimulate glutamine-dependent lipid synthesis. For this, we used HT29 cells in which SQOR or ETHE1 was knocked down using shRNA as previously described (19, 27). In comparison with HT29^scr^ control, the sulfide effect was significantly higher in SQOR knockdown cells (Fig. 1*E*). Basal radiolabel incorporation into lipids was significantly higher in the ETHE1 knockdown cells, whereas the magnitude of sulfide-induced stimulation was comparable with the HT29^scr^ controls.

To assess whether sulfide-stimulated labeling of lipids by glutamine is a general metabolic response, seven other cell lines were examined under normoxic (20% O_2_) *versus* hypoxic (2% O_2_) conditions (Fig. 1, *F* and *G*). With the exception of HepG2 and J774 cells, hypoxic conditions tended to increase radiolabeling of lipids compared with cells grown under normoxic conditions (Fig. 1*F*), which is consistent with the reported stimulation of reductive activation by hypoxia (28). Although the human hepatocellular carcinoma cells HepG2 were unresponsive to hypoxia, a decrease in lipid labeling was observed in J774 murine macrophage cells.

Like HT29^scr^ cells, sulfide increased lipid labeling in HCEC, HCT116, J774, 143B^wt^, and 143B^CytB^ cells (Fig. 1*G*). In contrast, sulfide decreased radiolabel incorporation in HepG2 cells. The wild-type osteosarcoma cybrid 143B^WT^ exhibited the highest (10- to 15-fold) sulfide-stimulated increase in radiolabel incorporation into lipids, whereas the 143B^CytB^ cybrids lacking an intact ETC exhibited only a small (10%), but statistically significant, increase. These results suggest an interplay between hypoxia and sulfide-based metabolic reprogramming, which leads to increased glutamine incorporation into lipids *via* activation of reductive carboxylation (Fig. 1*A*).

### Oxidative shifts in mitochondrial NAD(P)H pools inhibit sulfide-stimulated lipid biogenesis from glutamine

**Figure 2:**
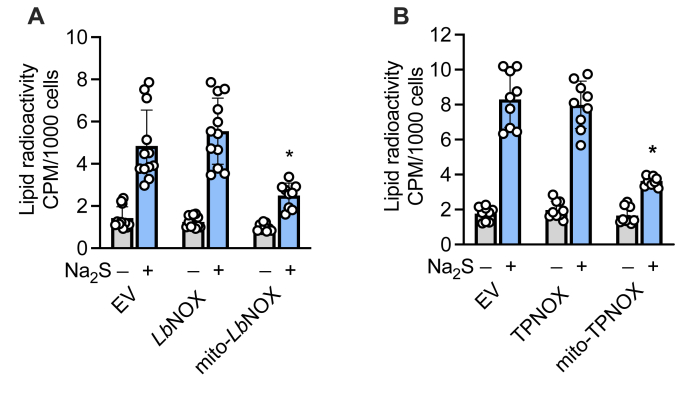
**Mitochondrial expression of *Lb*NOX or TPNOX decrease sulfide-activated lipid labeling from glutamine.***A* and *B*, Na_2_S (100 μM)-stimulated increase in radiolabel incorporation from [U-^14^C]-glutamine into lipids is suppressed in HT29 cells expressing mitochondrial but not cytoplasmic *Lb*NOX (*A*) or TPNOX (*B*). ∗Indicates significant difference (*p* < 0.0001) from sulfide-treated empty vector (EV) expressing cells or *Lb*NOX. Data represent the mean ± SD of 9 to 12 independent experiments.

Targeted dissipation of the cytoplasmic and mitochondrial NADH pools can be achieved by ectopic expression of the water-forming NADH oxidase, *Lb*NOX and *mito*-LbNOX (29), respectively. Sulfide stimulated similar levels of lipid labeling from glutamine in HT29 cells expressing the empty vector or cytoplasmic *Lb*NOX (Fig. 2*A*). In contrast, expression of *mito*-*Lb*NOX significantly decreased sulfide-stimulated radiolabeling of lipids by glutamine.

Next, we examined the effect of dissipating the cytoplasmic *versus* mitochondrial NADPH pool by ectopic expression of the water-forming NADPH oxidase (TPNOX, Fig. S1) (30). In contrast to expression of the empty vector or the cytoplasmic TPNOX, expression of *mito*-TPNOX significantly decreased sulfide activation of radiolabel incorporation from [U-^14^C]-glutamine to lipids (Fig. 2*B*).

### Dissipation of the mitochondrial NADH pool renders cells more resistant to respiratory poisoning by sulfide

**Figure 3:**
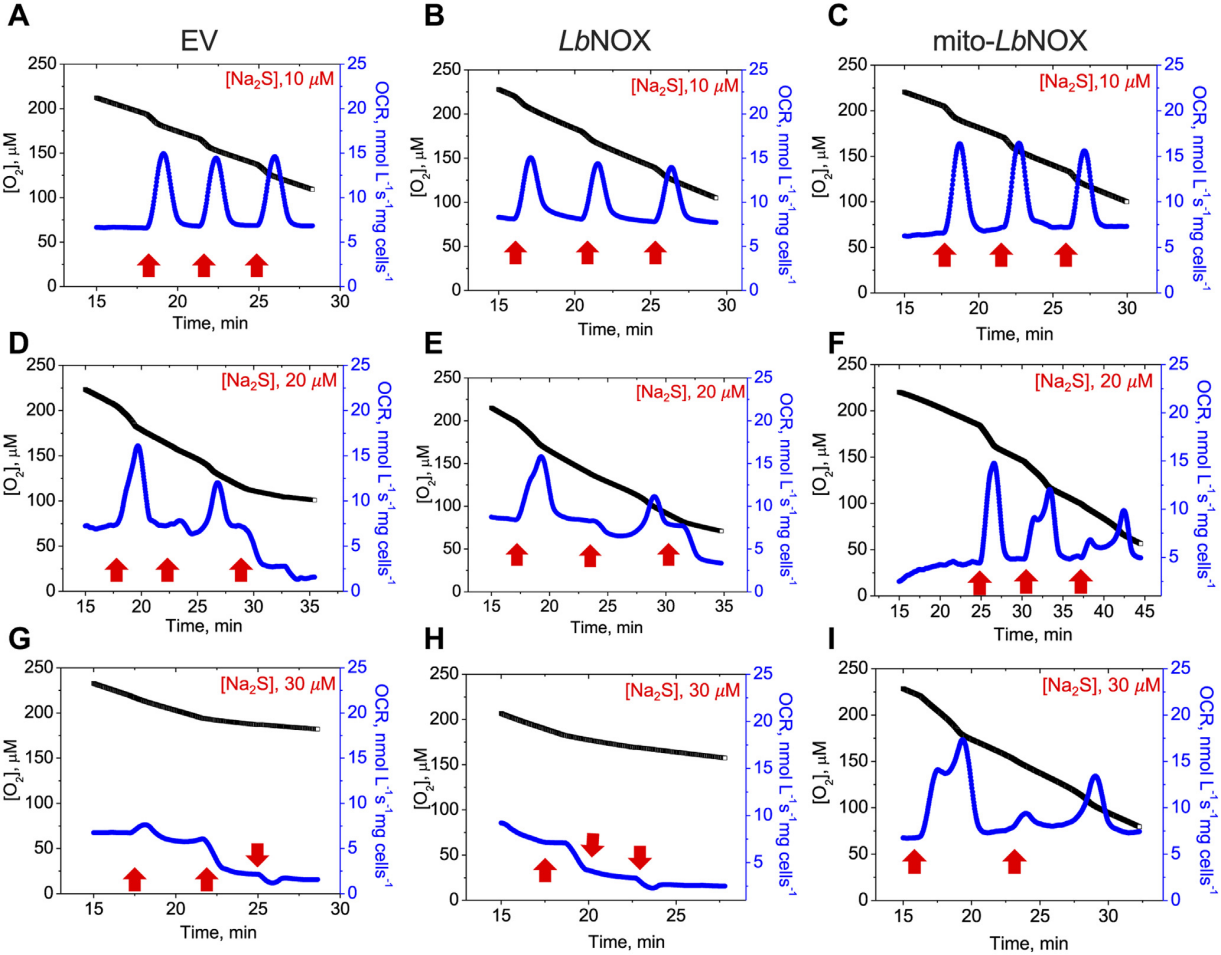
**Dissipation of the mitochondrial NADH pool increases sulfide tolerance.***A*–*C*, the differential effects of sulfide on oxygen consumption rate (OCR) in response to aliquots of Na_2_S added at the indicated times and concentrations (*red arrows*) to HT29 cells expressing empty vector (EV, *A*, *D* and *G*), cytoplasmic *Lb*NOX (*B*, *E* and *H*), or mitochondrial *Lb*NOX (*C*, *F* and *I*). The data are representative of three independent experiments.

To understand how the mitochondrial expression of *Lb*NOX affects sulfide metabolism, we examined the kinetics of oxygen consumption. At a relatively low concentration of sulfide (10 μM), HT29 cells expressing the empty vector, the cytoplasmic or mitochondrial form of *Lb*NOX, elicited similar responses, *i.e.*, an increase in the oxygen consumption rate (OCR), which returned to basal levels within ∼2 min (Fig. 3, *A*–*C*). At higher concentrations (≥20 μM), differences in the cellular response to sulfide were observed. In response to 20 μM sulfide (Fig. 3, *D*–*F*), cells expressing the empty vector or cytoplasmic *Lb*NOX showed similar responses with an increase in OCR following the first injection but signs of inhibition after the second. In contrast, *mito-Lb*NOX-expressing cells were more resistant to inhibition. At 30 μM sulfide, the differences were even more pronounced (Fig. 3, *G*–*I*). These data demonstrate that the mitochondrial NADH pool modulates sulfide-dependent OCR and are consistent with enhanced H_2_S clearance by cells expressing mitochondrial *versus* cytoplasmic *Lb*NOX (27).

### Sulfide elicits widespread lipidomic changes

**Figure 4:**
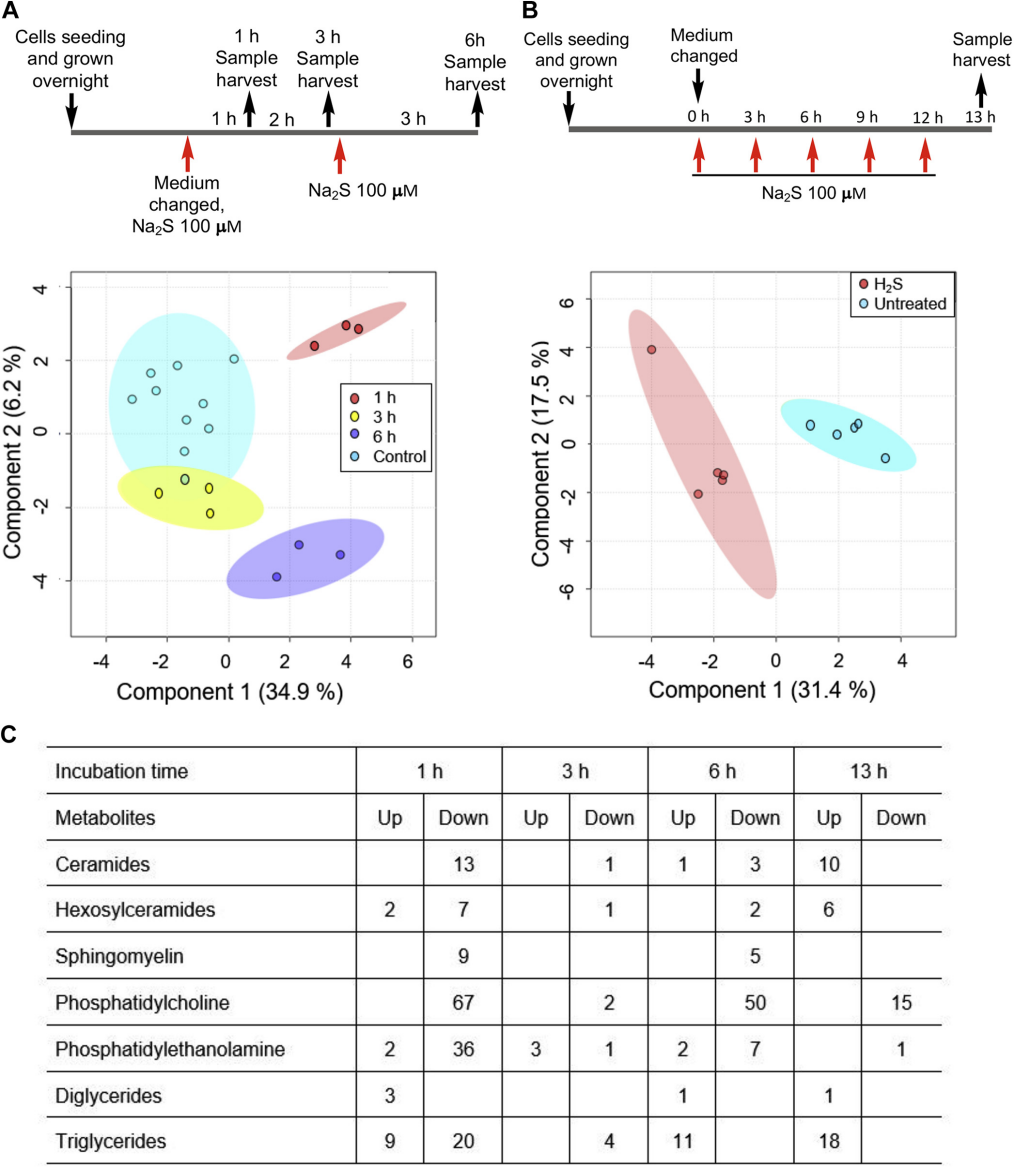
**Lipidomic analysis in H**_**2**_**S-treated HT29**^**scr**^**cells.***A* and *B*, partial least squares–discriminate analysis of the lipidomic data obtained with HT29^scr^ cells grown under hypoxic conditions (2% O_2_) and treated with Na_2_S (100 μM) at the indicated times as described in the schemes. The 95% confidence interval is indicated by the *elliptical pattern* per group. The data were sum normalized, log transformed, and mean centered. *C*, summary data showing classes of lipids that increased or decreased in response to sulfide treatment. A complete list of changes is presented in Table S1.

The sulfide-stimulated glutamate-dependent lipid labeling was ∼2-fold at 2% *versus* 3- to 4-fold at 20% oxygen in HT29^scr^ cells (Fig. 1*G*). Since colonocytes are exposed to an atmosphere with low oxygen tension, lipidomics analysis was conducted on HT29^scr^ cells cultured in an atmosphere of 2% oxygen. Multivariate analysis revealed significant perturbations in the distribution of lipids in HT29^scr^ cells 1 h after exposure to sulfide (Fig. 4*A*), which returned to control values after 3 h. Exposure to additional 100 μM doses of sulfide at 3 h intervals resulted in distinct patterns of changes (Fig. 4, *A* and *B*). After 1 h, lower levels of ceramides, sphingomyelin, and phosphatidylcholines were observed (Fig. 4*C* and Table S1). Hexosylceramides, phosphatidylethanolamine, and triglycerides on the other hand, showed a mixed response, with some species being up- and others, downregulated. At 13 h, the time point at which metabolic labeling studies were conducted (Fig. 1), ceramides (70%), hexosylceramides (75%), and triglycerides (140%) were the major lipid groups that were present at higher levels compared with untreated controls, whereas phosphatidylcholine (30%) was downregulated (Fig. 4*C*). At 13 h, we observed a 70% overall increase in the levels of those lipids that were differentially expressed between H_2_S treated *versus* untreated cells.

### Fatty acid synthesis inhibition decreases sulfide-induced lipid labeling

**Figure 5:**
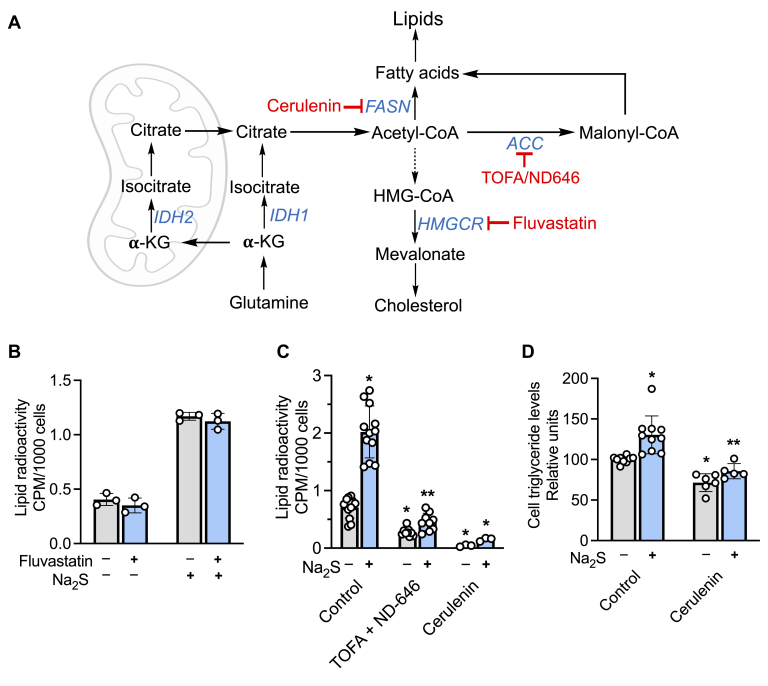
**Sulfide increases triglyceride levels.***A*, scheme showing the targets of inhibitors used to track the fate of glutamine-derived acetyl-CoA in fatty acid and cholesterol biosynthesis. ACC, FASN, and HMGCR denote acetyl-CoA carboxylase, fatty acid synthase, and 3-hydroxy-3-methylglutaryl-coenzyme A reductase, respectively. *B* and *C*, radiolabel incorporation from [U-^14^C]-glutamine into lipids was not affected by fluvastatin, which inhibits cholesterol synthesis, but was significantly diminished in the presence of acetyl-CoA carboxylase inhibitors (TOFA + ND-646 at 10 and 0.5 μM, respectively) and the fatty acid synthase inhibitor, cerulenin (20 μM). ∗, ∗∗Denote statistically significant differences from untreated or Na_2_S-treated controls, respectively, *p* < 0.001. *D*, HT29^scr^ cells treated with Na_2_S (100 μM) showed an increase in triglyceride levels after 13 h, which was attenuated in the presence of cerulenin (20 μM). ∗, ∗∗Denote statistically significant differences from untreated or Na_2_S-treated controls, respectively, *p* < 0.003. Data represent the mean ± SD of 3 to 12 independent experiments.

Glutamine-derived acetyl-CoA can support fatty acid and cholesterol synthesis (Fig. 5*A*). Since cholesterol is not picked up in our lipidomics analysis, we examined whether inhibition of cholesterol synthesis by fluvastatin, a 3-hydroxy-3-methyl-glutaryl CoA reductase inhibitor (31), decreases radiolabel incorporation from glutamine. However, no change in lipid labeling was observed in the presence of fluvastatin (Fig. 5*B*), whereas the fatty acid synthase inhibitor cerulenin (32) and the acetyl-CoA carboxylase inhibitors, TOFA (33) and ND-646 (34), inhibited sulfide-activated lipid labeling as expected (Fig. 5*C*).

### Sulfide increases triacylglyceride biosynthesis

To assess whether the increase in metabolic labeling from glutamine to lipids was accompanied by a net increase in lipid biogenesis, we measured triglycerides levels directly, owing to the relative ease with which this lipid group can be detected. Sulfide treatment resulted in a 30 ± 23% (n = 9, *p* < 0.003) increase in the triglyceride pool in HT29^scr^ cells, which was attenuated by pharmacological inhibition of fatty acid synthase with cerulenin (Fig. 5*D*).

## Discussion

In comparison with the hundreds of protein targets of persulfidation that have been identified and ascribed to H_2_S signaling (35, 36), little is known about how H_2_S influences metabolism (4). Intriguing connections between H_2_S and lipid synthesis have been described in the literature ranging from the H_2_S-induced increase in lipid accumulation in *Nanochloropsis oceanica* for microalgal biodiesel production (37) to cysteine/H_2_S being pro-obesogenic (reviewed in (21)). In this study, we have elucidated the carbon source used for lipid synthesis by cultured cells treated with H_2_S and demonstrate that it is linked to the inhibitory effect of H_2_S on the ETC. The consequent pleiotropic effects on cell metabolism appear to prioritize glycolysis for ATP synthesis as described previously (27) and shifts operation of the tricarboxylic acid cycle (TCA) cycle in the reductive direction for redox recycling and macromolecular synthesis (19).

**Figure 6:**
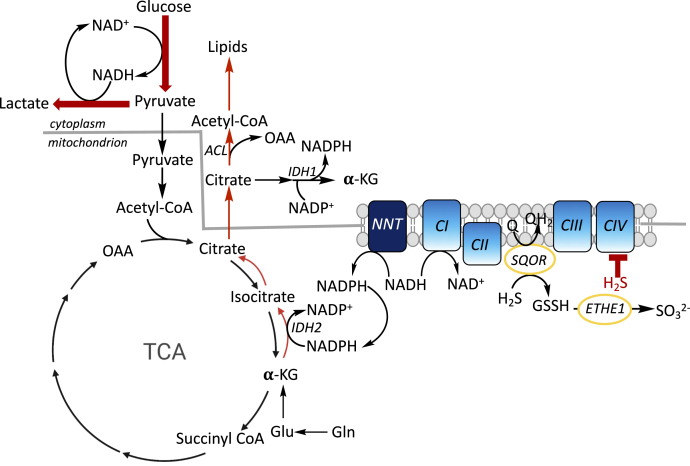
**Summary of the metabolic effects of H**_**2**_**S.** SQOR oxidizes H_2_S to GSSH, which is subsequently converted to sulfite by ETHE1. If H_2_S builds up, complex IV is inhibited, leading to a build-up of NADH relative to NAD^+^. Under these conditions, cellular ATP needs are met *via* increased aerobic glycolysis and lactate production (*thick red arrows*) as reported previously (27). Carbon for macromolecular synthesis including lipids is derived from glutamine *via* reductive carboxylation of α-ketoglutarate, leading to citrate. Conversion of glutamine to α-ketoglutarate can occur in the cytoplasm and the mitochondrion and, for simplicity, is shown in only one compartment. The transhydrogenase NNT can catalyze the transfer of reducing equivalents from NADH to NADP^+^, furnishing NADPH to drive IDH2-catalyzed reductive carboxylation. ATP citrate lyase (ACL) cleaves citrate to acetyl-CoA, which is incorporated into lipids (*thin red arrows*). OAA and α-KG are oxaloacetate and α-ketoglutarate, respectively.

We have recently demonstrated that H_2_S stimulates aerobic glycolysis and enhances lactate production (Fig. 6) (27). Under these conditions, glycolysis presumably functions primarily to support cellular ATP needs, with a redox neutral cycle being established between NAD^+^ reduction by glyceraldehyde 3-phosphate dehydrogenase and NADH oxidation by lactate dehydrogenase. By inhibiting the ETC, H_2_S decreases the NAD^+^/NADH ratio (19), disfavoring the oxidative TCA cycle. The anabolic needs of the cell are presumably met by glutamine metabolism under these conditions. Consistent with this model, reductive carboxylation is stimulated by H_2_S as evidenced by enhanced [^13^C]-glutamine-derived labeling of citrate and other metabolites (19). In this study, we demonstrate that H_2_S stimulates lipogenesis using glutamine carbon in a metabolic signaling process that begins in the mitochondrion and is sensitive to the NAD(P)H pool in this compartment (Fig. 6).

Nicotinamide nucleotide transhydrogenase uses the transmembrane proton gradient to catalyze electron transfer from NADH to NADP^+^. An increase in mitochondrial NADH is predicted to increase the levels of NADPH, driving NADPH-dependent isocitrate dehydrogenase (IDH). The latter converts α-ketoglutarate derived from glutamine to isocitrate. Knockdown of nicotinamide nucleotide transhydrogenase inhibits reductive carboxylation (38). Isocitrate is subsequently isomerized to citrate, which is converted by ATP citrate lyase to acetyl-CoA and oxaloacetate (Fig. 6). Both the cytoplasmic and mitochondrial IDH isoforms, IDH1 and IDH2, respectively, contribute to reductive carboxylation-dependent lipogenesis in cells with mitochondrial dysfunction (39). Although the relative importance of the IDH isoforms was not addressed in our study, the sensitivity of H_2_S-stimulated lipogenesis to the mitochondrial NAD(P)H pool (Fig. 2) suggests the importance of the IDH2 (Fig. 6).

An observation that is not explained by our model is why mitochondrial, but not cytoplasmic, TPNOX expression attenuated lipid labeling since fatty acid synthesis requires NADPH. In addition to the well-characterized cytoplasmic fatty acid synthase, eukaryotes also have a mitochondrial pathway that furnishes octanoate for lipoic acid synthesis and acylated acyl carrier proteins that are important for the assembly of ETC components (40). A contribution of the mitochondrial fatty acid synthesis pathway to H_2_S-stimulated lipogenesis is, however, not supported by the lipidomics data, which reveal changes across several classes of lipids.

As discussed earlier, H_2_S stimulates aerobic glycolysis leading to the stoichiometric conversion of glucose to two lactates, restricting pyruvate availability for the TCA cycle. Mitochondrial expression of TPNOX is expected to stimulate the conversion of NADH to NADPH, thus reducing competition from complex I for the CoQ pool as shown previously with mito-*Lb*NOX (27). In this scenario, H_2_S oxidation *via* SQOR and the ETC would be more efficient, explaining reduced lipid biogenesis from glutamine (Fig. 2*B*), which is consistent with the sensitivity of this process to H_2_S concentration (Fig. 1*D*).

The lipidomics data (Table S1) and triglyceride analysis (Fig. 5*D*) revealed that H_2_S induced a net increase in the synthesis of certain lipids while decreasing levels of others compared with untreated controls. We posit that lipid biogenesis helps alleviate the reductive shift in the pyridine nucleotide pool induced by H_2_S. In other words, lipid synthesis could represent an adaptive cellular strategy for recycling NAD(P)H to NAD(P)^+^ to alleviate reductive stress. Furthermore, it is possible that, in addition to serving as a carbon source, citrate shuttles reducing equivalents from the mitochondrion to the cytoplasm. Oxidation of citrate-derived isocitrate to α-ketoglutarate by cytoplasmic IDH1 would generate NADPH (Fig. 6).

It is interesting to note that metabolic adaptation to hypoxia and H_2_S exhibit overlapping signatures. Like H_2_S, hypoxia increases glucose uptake (28), aerobic glycolysis, and reductive carboxylation (28, 41) as well as glutamine-dependent lipid synthesis (42). In both cases, the changes originate in the ETC either due to O_2_ limitation (hypoxia) or complex IV inhibition (H_2_S) and lead to an integrated metabolic reprogramming in the mitochondrial and cytoplasmic compartments. The similarities between hypoxic and H_2_S-induced reprogramming warrant further study.

In summary, we have demonstrated that H_2_S stimulates lipogenesis in a nonmalignant and several malignant cell lines. We have identified glutamine as the carbon source for lipogenesis that is made available *via* reductive carboxylation, which, in turn, is driven by inhibition of the ETC by H_2_S. This study provides a potential explanation for cell-based and population studies that have reported a correlation between H_2_S/cysteine and lipid synthesis.

## Experimental procedures

### Materials

Na_2_S, nonahydrate (99.99% purity), glucose, hydrocortisone, insulin, apo-transferrin, sodium selenite, sodium orthovanadate, uridine (cell culture grade), Protease Inhibitor Cocktail for use with mammalian cell and tissue extracts, puromycin, cerulenin, fluvastatin sodium hydrate, doxycycline, RIPA buffer, HPLC grade tert-Butyl methyl ether (MTBE), and chloroform were from Sigma. TOFA and ND-646 were from MedChemExpress. Dulbecco's modified Eagle's medium (DMEM) (with 4.5 g/l glucose, glutamine, and 110 mg/l sodium pyruvate), RPMI 1640 with glutamine, medium 199, fetal bovine serum (FBS), penicillin/streptomycin mixture, trypsin, EDTA, PBS, and Dulbecco's phosphate-buffered saline medium (DPBS) were from Gibco. Geneticin was purchased from Life Technologies. Hepes and LC-MS grade acetonitrile, methanol, water, isopropanol, and ammonium formate were from Fisher, and Eagle's minimal essential medium (EMEM) was from Lonza. [U-^14^C]-glucose (263.0 mCi/mmol) and [U-^14^C]-glutamine (281.0 mCi/mmol) were from PerkinElmer. EquiSPLASH lipid standard (#330731) was purchased from Avanti Polar Lipids, Inc.

### Cell lines and culture conditions

Human colon cancer cell lines HT29, HCT116, and DLD1; human embryonic kidney cells HEK293; human hepatocarcinoma cells HepG2; and murine macrophage cells J774 were obtained from American Type Culture Collection. The concentrations of glucose in the culture media were as follows: 11.1 mM (RPMI 1640), 25 mM (DMEM), 5.56 mM (199 and EMEM), and 21.1 mM in the culture medium for HCEC cells (a mixture of 199 and DMEM 1:4 v/v). The glutamine concentrations in the culture media were as follows: 2.05 mM (RPMI 1640), 2.63 mM (DMEM), 0.68 mM (199), 2.0 mM (EMEM), and 2.24 mM in the culture medium for HCEC cells.

143B^WT^ and 143B^Cytb^ cybrids were a generous gift from Dr Matthew Vander Heiden (MIT), and the nonmalignant human colon cell line HCEC was obtained from Dr Eric Fearon (University of Michigan).

HCT116, DLD1, HEK293, and J774 cells were cultured in DMEM supplemented with 10% FBS, 100 units/ml penicillin, and 100 μg/ml streptomycin. HepG2 cells were cultured in EMEM medium supplemented with 10% FBS, 100 units/ml penicillin, and 100 μg/ml streptomycin. HT29 cells were cultured in RPMI 1640 medium supplemented with 10% FBS, 100 units/ml penicillin, and 100 μg/ml streptomycin.

HT29^scr^, SQOR, and ETHE1 knocked down cells were described (19, 27) and were cultured as described above for HT29 cells except that the culture medium was supplemented with 1 μg/ml puromycin.

With the exception of HCEC, all other cell lines were grown in humidified cell culture incubators at 37 °C containing a 5% CO_2_ atmosphere. HCEC cells were cultured in a mixture of DMEM and medium 199 (4:1 v/v), supplemented with 100 units/ml penicillin, 100 μg/ml streptomycin, 20 ng/ml human recombinant epidermal growth factor (Gibco), 2% (^v^/_v_) cosmic calf serum (Hyclone), 1 μg/ml hydrocortisone, 10 μg/ml insulin, 2 μg/ml apo-transferrin, 5 nM sodium selenite, in a humidified hypoxic incubator with an atmosphere containing 2% O_2_, 5% CO_2_, and 93% N_2_ at 37 °C.

### Stable expression of cytoplasmic and mitochondrial LbNOX and TPNOX

Stable expression of *Lb*NOX in HT29 cells has been described (27). Stable expression of TPNOX was achieved using the pLVX-TRE3G empty vector, TPNOX, mito-TPNOX, and pLVX TET ON (generously provided by Dr Valentin Cracan (Scintillon Institute)). Lentiviral packaging of these vectors was performed at the Vector Core (University of Michigan). HT29 cells (25,000 cells per well) were seeded into 12-well plates containing 1 ml RPMI 1640 medium supplemented with 10% FBS, 100 units/ml penicillin, and 100 μg/ml streptomycin per well. Following incubation in a 5% CO_2_ incubator for 24 h at 37 °C, the cells were transduced with optimized viral titers for 24 h. Then, the culture medium was replaced with fresh virus-free medium and the incubation was continued for 24 h. Cells were then selected using medium containing 500 μg/ml geneticin and 1 μg/ml puromycin. Confluency in 10-cm plates was reached in ∼2 to 3 weeks during which time cells were cultured in RPMI 1640 medium supplemented with 10% FBS, 100 units/ml penicillin, 100 μg/ml streptomycin, 300 μg/ml geneticin (Life Technologies), and 1 μg/ml puromycin. TPNOX expression was induced 24 h prior to the start of an experiment using 300 ng/ml doxycycline.

Expression of TPNOX was validated in induced cells that were washed twice with PBS and lysed in RIPA buffer with 10 μl/ml protease inhibitor cocktail for mammalian cell extracts. The lysates were treated to three freeze–thaw cycles and centrifuged for 5 min at 12,000*g* at 4 °C, and the protein concentration in the supernatant was determined using the Bradford reagent (Bio-Rad). TPNOX (which is FLAG tagged) was detected using an anti-FLAG antibody (Sigma, F1804) at a dilution of 1:2000 and horseradish peroxidase–linked anti-mouse IgG as a secondary antibody (GE Healthcare, NA931V) at a dilution of 1:20,000. The KwikQuant Western Blotting Substrate (Kindle Biosciences) was used to develop the membranes, which were analyzed using the KwikQuant imager (Kindle Biosciences).

### Metabolic labeling of lipids from [U-^14^C]-glucose or [U-^14^C]-glutamine

Cells (7 × 10^5^/well) were seeded in six-well plates containing 2 ml culture medium and incubated overnight at 37 °C in a humidified 5% CO_2_ atmosphere. On the next day, the old medium in each well was replaced with fresh culture medium containing 0.1 μCi per ml of [U-^14^C]-glucose or [U-^14^C]-Gln. The specific radioactivity of [U-^14^C]-glucose and [U-^14^C]-Gln in RPMI medium was 9 and 48.8 μCi/mmol, respectively. The specific radioactivity of [U-^14^C]-glucose in the culture medium for HCEC cells was 4.7 μCi/mmol. Na_2_S from a freshly prepared stock solution (100 mM in water) was added to a final concentration of 100 μM except in experiments where the sulfide concentration dependence was determined. Untreated controls were maintained in separate culture plates to avoid cross-contamination with H_2_S. Fresh aliquots of Na_2_S were added at 3, 6, 9, and 12 h using freshly prepared Na_2_S stock solutions each time (Fig. 1*B*). During the treatment, control plates were also taken out and then returned to the incubator along with the treated plates. One hour after the last sulfide treatment (*i.e.*, at 13 h), the medium was aspirated, the cells were washed twice with 2 ml ice-cold PBS, and frozen by placing the covered plates on dry ice and storing them at −20 °C. When used, lipid biosynthesis inhibitors (fluvastatin, cerulenin, TOFA, and ND-646) dissolved in dimethyl sulfoxide were added immediately after the cells were seeded and present through the entire duration of the experiment. Control samples received an equivalent volume of DMSO.

### Analysis of cell lipid radioactivity

Lipids were extracted by adding 500 μl of a solution containing three volumes of hexane and two volumes of isopropanol to each well and mixed by repeated (5–6 times) pipetting. The sample was then transferred to a 1.7-ml Eppendorf tube containing 300 μl PBS. Then, a second 500-μl aliquot of the hexane-isopropanol mixture was added to wash out any remaining sample and collected in the same tube. The samples were vortexed vigorously and centrifuged for 5 min at 13,000*g* at 25 °C, and the upper layer from each sample tube was transferred to a new 1.7-ml tube containing 300 μl PBS, and the extraction process was repeated. Following the second centrifugation, the upper layer from each tube was carefully transferred into vials containing liquid scintillation cocktail and radioactivity was measured.

### Lipidomics analysis

#### Sample preparation

HT29^scr^ cells (10^6^ cells in 2 ml medium/well) were grown overnight in six-well plates at 37 °C in a humidified 5% CO_2_ atmosphere containing 2% O_2_ in nitrogen. On the next day, the medium was replaced by fresh culture medium and 100 μM Na_2_S was added every 3 h as described in the metabolic labeling schemes in Figure 4, *A* and *B*. Samples were collected at 1, 3, 6, and 13 h following addition of the first aliquot of sulfide at time 0. For ease of sample handling, controls *versus* 1, 3, and 6 h Na_2_S treatment were obtained from one experiment. The 13 h sample was obtained in a separate experiment with an independent control. Each sample was run with 3 to 5 replicates.

At the desired time, the medium was aspirated and the cells were washed once with 2 ml PBS. Then, 0.5 ml per well of 0.05% trypsin solution with EDTA was added and the plates were incubated for 8 to 10 min at 37 °C. The trypsinized cells were suspended in 0.5 ml of culture medium, and the suspension was centrifuged in 1.5-ml sample tubes for 5 min at 1700*g* at 4 °C. The supernatant was aspirated, 1 ml of cold PBS per tube was added, vortexed, and centrifuged, and the supernatant was removed. Finally, 0.1 ml of cold PBS was added to each cell pellet, vortexed, and frozen on dry ice and stored at −80 °C until use.

#### Lipid extraction

Total lipid extracts from the cell pellets were prepared using MTBE liquid–liquid extraction. Briefly, 400 μl ice-cold methanol was added to the cell pellet followed by 30 s of sonication and 30 s of vortex mixing. Then, 10 μl of the internal standard (EquiSplash [#330731], Avanti Polar Lipids) was added to the samples followed by the addition of 500 μl MTBE. The mixture was incubated at 4 °C for 1 h with 650 rpm shaking. Water (500 μl) was then added followed by vortex mixing and incubated at 4 °C for 15 min with 650 rpm shaking. The samples were centrifuged at 8000*g* for 8 min at 4 °C to induce phase separation. The upper (organic) layer was transferred and stored on ice. MTBE (200 μl) was added to the lower (aqueous) phase followed by vortex mixing (30 s) and incubation at 4 °C for 15 min with 650 rpm shaking. The upper (organic) phase was removed and combined with the first extraction. The combined organic layers were dried with nitrogen at 30 °C, and the dried lipid extract was resuspended in 200 μl of chloroform:methanol (1:1, v/v) with 200 μM of butylated hydroxytoluene and stored at −20 °C. Prior to analysis, extracts were further diluted 1:5 in acetonitrile:isopropanol:water (1:2:1, v/v/v). The lower aqueous phase was used to determine the protein content using a BCA kit (bicinchoninic acid assay, Thermo Fisher Scientific).

#### Lipid analysis

Ultra-Performance Liquid Chromatography (UPLC) Data Independent Tandem Mass Spectrometry with Traveling Wave Ion Mobility (HDMS^E^) was used for lipid analysis. UPLC was performed on a Waters ACQUITY UPLC system. The separation was achieved using a Kinetic HILIC (2.6 μm; 2.1 × 150 mm) column (Phenomenex). The mobile phase A was 10 mM ammonium formate in water/acetonitrile (5:95, v/v) and mobile phase B was 10 mM ammonium formate in water/acetonitrile (50:50, v/v). The gradient was ramped from 0.1% to 20% B in 10 min, ramped to 80% B in 0.1 min, held at 80% B for 2.9 min, ramped to 0.1% B in 0.1 min, and held for 2.9 min. The flow rate was 0.5 ml/min. The column was maintained at 30 °C, and the auto-sampler was kept at 5 °C. A 2-μl injection was used for all samples. HDMS^E^ experiments were performed with a traveling wave ion mobility–enabled hybrid quadrupole orthogonal acceleration time-of-flight mass spectrometer (SYNAPT G2-S, Waters Corporation). HDMS^E^ parameters were adopted with slight modifications (43). The capillary voltage was 2.0 kV, and sampling cone voltage was 30 V. Nitrogen at a flow of 650 l/h was used as the desolvation gas with a constant desolvation temperature of 400 °C. The source temperature was set at 125 °C. Data were acquired over the *m/z* range of 100 to 1800. The mass spectrometer was operated in ion mobility, data independent (MS^E^) acquisition for both positive and negative ion modes. Argon gas was used for collision-induced dissociation. Leucine enkephalin (0.1 mg/ml) at a flow rate of 7.5 μl/min was used as the lock-mass to ensure high mass accuracy data acquisition. Data were acquired with MassLynx v4.1 (Waters).

#### Data processing/bioinformatics

UPLC-HDMS^E^ data were analyzed with MS^E^ Data Viewer v1.2 (Waters), DriftScope HDMS v2.7 (Waters), Progenesis QI v2.2 (Nonlinear Dynamics), MetaboAnalyst 3.0 (44, 45), and Prism 6 (GraphPad). Raw data files were directly imported into Progenesis QI where retention time alignment, peak picking, deconvolution of adducts, relative abundance, and preliminary identification were performed. Preliminary identification involved accurate mass correlation at a threshold of 10 ppm to LIPIDMAPS (http://www.lipidmaps.org). The processed data generated from Progenesis QI, which included peak area and *m/z* value, were exported into MetaboAnalyst for multivariate analysis, which included principal component analysis and partial least square discriminate analysis. Univariate analysis *via* Prism 6 was performed using normalized values generated from Progenesis QI. Putative and confirmatory structure assignments relied on chromatographic retention time, HDMS, and positive and negative ion mass spectral correlation.

### Measurement of triglyceride levels

HT29^scr^ cells (10^7^ cells per 10-cm plate) were seeded in 10 ml RPMI 1640 medium supplemented with 10% FBS, 100 units/ml penicillin, 100 μg/ml streptomycin, and 1 μg/ml puromycin and cultured overnight with cerulenin (20 μM) dissolved in DMSO or with an equivalent volume of DMSO. The cells were cultured at 37 °C in a humidified cell culture incubator with an atmosphere of 5% CO_2_ in air. On the next day, fresh medium containing cerulenin or DMSO together with Na_2_S (100 μM) was added to half the plates. At 3, 6, 9, and 12 h, freshly prepared Na_2_S (100 μM) was added to the treatment plates. Samples for triglycerides analysis were prepared 1 h after the last treatment, *i.e.*, at 13 h. For this, the medium was aspirated and the cells were washed twice with 8 ml of ice-cold PBS and then scraped in 1 ml PBS. The cell suspension was transferred to preweighed sample tubes and centrifuged for 5 min at 1700*g* at 4 °C, and the supernatant was removed. The tube was weighed again to determine the pellet mass. Next, the cell pellets (∼40–50 mg wet weight) were suspended in 1 ml 5% Nonidet P40 substitute solution (Fluka). Triglycerides in the samples were measured using the Triglyceride Quantification Kit (Sigma) as per the manufacturer’s protocol. A control in which the assay buffer substituted for lipase was included, and the background value was subtracted from all readings. In addition, some samples were spiked with a known concentration of a triglyceride standard to ensure accuracy of the method. The triglyceride levels were normalized to cell pellet weight.

### Oxygen consumption rate measurements

OCR measurements on HT29 cells expressing an empty vector or cytoplasmic or mitochondrial *Lb*NOX were made using a respirometer (Oroboros Instruments Corp). Cells were cultured to a confluency of 80% to 90% in 10-cm plates, and the expression of *Lb*NOX was induced by 24 h culture with 300 ng/ml doxycycline in the medium. Following removal of culture medium by aspiration, the cells were washed twice with cold PBS and trypsinized (0.05% trypsin in PBS). The cells were suspended in 10 ml of cell culture medium and centrifuged at 1700*g* for 5 min at 4 °C. The supernatant was discarded, and the cell pellet was suspended in 1 ml modified DPBS (containing 100 mg/l CaCl_2_ and 100 mg/l MgCl_2_•6H_2_O) supplemented with 5 mM glucose and 20 mM Hepes, pH 7.4 and centrifuged at 1700*g* for 5 min at 4 °C in a preweighed sample tube. After discarding the supernatant, the tube was weighed to estimate the cell mass. The cell pellet was suspended in modified DPBS to give a 5% suspension (^w^/_v_) and stored on ice for up to 1.5 h. Just before the OCR measurement, an aliquot of the 5% suspension was diluted with modified DPBS to make 2 ml of a 1% suspension and placed in the respirometer chamber, and the OCR was allowed to stabilize over ∼15 to 20 min at 37 °C with constant stirring at 750 rpm. Then, Na_2_S (from a freshly prepared 10 mM stock in water) was injected to give the desired final concentration (10, 20, or 30 μM) per injection.

## Data availability

All data are contained within the article and in the supplemental section.

## Supporting information

This article contains supporting information.

## Conflict of interest

R. B. is a paid member of the scientific advisory board of Apneo Therapeutics and owns equity in the company.
