# Supplementary material for: Hydrogen sulfide stimulates lipid biogenesis from glutamine that is dependent on the mitochondrial NAD(P)H pool
Source: J Biol Chem. 2021 Jul 10;297(2):100950. doi: 10.1016/j.jbc.2021.100950 (PMC8342795; doi:10.1016/j.jbc.2021.100950)
Supplement: Supplemental Figure S1 [file mmc2.docx]

**SUPPLEMENTAL INFORMATION**

**Hydrogen sulfide stimulates lipid biogenesis from glutamine that is dependent on the mitochondrial NAD(P)H pool**

Sebastian Carballal^1,2†^, Victor Vitvitsky^1†^, Roshan Kumar^1^, David A. Hanna^1^, Marouane Libiad^1^, Aditi Gupta^1^, Jace W. Jones^3^ and Ruma Banerjee^1^*

^1^Department of Biological Chemistry, Michigan Medicine, University of Michigan, Ann Arbor, Michigan 48109

^2^Departamento de Bioquímica, Facultad de Medicina and Centro de Investigaciones Biomédicas (CEINBIO), Universidad de la República, Montevideo, Uruguay, ^3^Department of Pharmaceutical Sciences, University of Maryland School of Pharmacy, Baltimore, MD 21201

*Corresponding Author. Email: [rbanerje@umich.edu](mailto:rbanerje@umich.edu)

**Table of Content**

**Figure S1. Expression of TPNOX and *mito-*TPNOX in HT29 cells**

**Table S1. Lipidomic data**


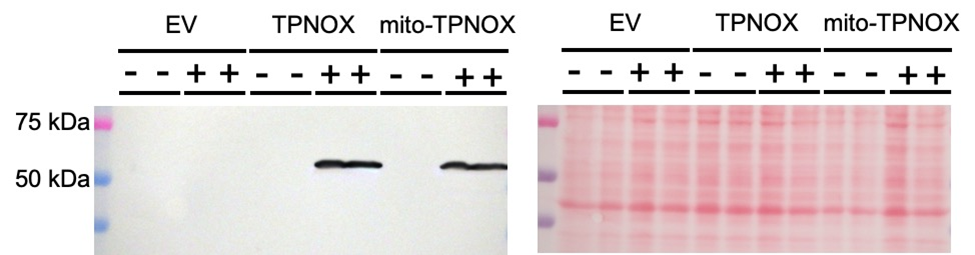


**Figure S1. Expression of TPNOX and *mito-*TPNOX in HT29 cells.** Western blot analysis of cell lysates 24 h after ± 300 ng/ml doxycycline (*left*) and equal loading of the same membrane as visualized by Ponceau S staining (*right*).

**Table S1.** Lipidomic data for Figs. 4A and 4B. **Tab 1-4**. List of lipids that reached statistical significance in H_2_S treated HT29 cells versus controls at 1, 3, 6 and 13 h, respectively. Column headings are as follows: experimental *m/z* values are the experimentally determined precursor ion *m/z* value; lipid ID is the identification of the lipid based on experimental data (accurate *m/z* of precursor ion, chromatographic retention ion, ion mobility, and tandem mass spectrometry); anova p-values were determined via Progenesis QI software using anova tests; ppm is parts per million and represent the mass accuracy of the measured precursor ion; highest mean is the group which had the highest abundance; adduct represents which adduct the precursor ion was identified as; t_R_ (min) is the chromatographic retention time in minutes; mean is the averaged normalized signal abundance for each *m/z* value in the respective group; St Dev is the standard deviation of the normalized signal; % CV is the percent coefficient of variation of the normalized signal. The control group for 1-, 3-, and 6-hour comparisons had 10 biological replicates (n=10) and each hour cohort (1-, 3-, and 6-hour) had 3 biological replicates (n=3). The control versus 13-hour comparison had n=5 for both the control and 13-hour cohort. Lipid abbreviations are as follows: Cer=ceramide, HexCer=hexosyl ceramide, SM=sphingomyelin, PC=glycerophosphocholine, PE=glycerophosphoethanolamine, LPC=lysophosphocholine, LPE=lysophosphoethanolamine, DG=diacylglycerol, and TG=triacylglycerol.
